# HLA-DQ and alcohol in the pathogenesis of irritable bowel syndrome in college students: a case–control study

**DOI:** 10.1038/s41598-023-40295-2

**Published:** 2023-08-10

**Authors:** Huaying Liu, Lan Huang, Li Li, Tingting Lu, Huiping Liang, Chunbin Liu

**Affiliations:** 1 Department of Medicine, Guangxi Medical College, No 8 Kunlun Road, Nanning, 530023 Guangxi China; 2 Dean’s Office of Guangxi Medical College, No 8 Kunlun Road, Nanning, 530023 Guangxi China; 3 Department of Medical Technology, Guangxi Medical College, No 8 Kunlun Road, Nanning, 530023 Guangxi China; 4 Department of Internal Medicine, The Fifth Affiliated Hospital of Guangxi Medical University, Nanning, 530022 Guangxi China

**Keywords:** Genetics, Functional gastrointestinal disorders

## Abstract

Some researchers have shown that genetics contribute to the incidence of IBS. However, no research has focused on the interaction between HLA-DQ and living habits in the pathogenesis of IBS. The present study explored the risk factors for IBS in college students of Guangxi Han nationality and explored the interaction between HLA-DQ and living habits on the pathogenesis of IBS. Univariate and multivariate analyses were used to determine the risk factors for IBS. Logistic interaction analysis and the Excel table made by Andersson were used to explore the interaction between genes and living habits in the context of IBS. We found that low expression of HLA-DQ2 and DQ8 were associated with the pathogenesis of IBS, while mild to moderate alcohol consumption was associated with the occurrence of IBS symptoms. Only the HLA-DQ8 gene and alcohol consumption had additive interactions in the context of the occurrence of IBS. In other words, for college students of Guangxi Han nationality, HLA-DQ2 and HLA-DQ8 might be protective against IBS, while alcohol consumption might be an independent risk factor. There was an additive interaction between HLA-DQ8 and alcohol consumption in the occurrence of IBS.

## Introduction

Irritable bowel syndrome (IBS) is the most common type of functional gastrointestinal disease (FGID), which is characterized by abdominal pain and is often accompanied by changes in defecation frequency or faecal properties^1,2^. According to the literature, the prevalence of IBS varies greatly among different countries. The combined prevalence of all studies using the Rome III standard was 9.2%, while that using the Rome IV standard was 3.8%^3–5^. In China, the prevalence of IBS is higher than that in other Asian countries^6^. Since IBS has the characteristics of recurrent attacks and the current treatment for IBS involves only symptom management, the quality of life and work efficiency of IBS patients are seriously affected^7–9^. Clarifying the pathogenesis of IBS is of great significance to prevent the occurrence of IBS and improve the therapeutic effect. However, the aetiology and mechanism of IBS are still not completely clear. IBS is the result of multiple factors, such as abnormal gastrointestinal dynamics, visceral hypersensitivity, abnormal nervous system, intestinal infection, intestinal microecological imbalance and mental disorder^10–12^. In addition, relevant studies on the family aggregation of IBS and the incidence of IBS in twins in the early twenty-first century indicated that genetic factors may play a role in the pathogenesis of IBS^13^. Research on IBS susceptibility genes is helpful to carry out personalized prevention and treatment, which can improve the prognosis of IBS^14^.

Human leukocyte antigen (HLA) is the first discovered gene cluster closely related to the human immune response^15^. At present, some studies have suggested that the HLA-DQ gene is correlated with the pathogenesis of IBS. They supported that different populations had different IBS susceptibility genes^16–20^. However, the susceptibility gene for IBS in Guangxi has not been reported. Since IBS has a complex pathogenesis, genetic susceptibility is only one of the pathogenic factors. The environment, diet, psychology and other factors can also cause pathophysiological changes, leading to the occurrence of IBS^12,21^. The aim of this study was to analyse the effects of the HLA gene and different living habits on the occurrence of IBS in college students of Guangxi Han nationality and to provide new ideas for understanding the aetiology of IBS and developing methods for the individualized treatment of IBS.

## Methods

### Ethics approval

The study was approved by the Ethics Committee of Guangxi Medical College. All the subjects were informed of the details of the study and signed the informed consent form.

### Inclusion criteria and exclusion criteria

The paired experimental subjects were college students of Guangxi Han nationality who were recruited in March 2021. All of the students were sophomores majoring in clinical medicine and had been enrolled for at least one year. Their curriculums were consistent. The inclusion criteria were as follows: (1) patients met Rome IV criteria and were diagnosed with IBS by gastroenterologists at the First Affiliated Hospital of Guangxi Medical University (International Classification of Diseases, Eleventh Revision, Clinical Modification, code DD91.0); (2) healthy volunteers of the same sex, an age ± 1 year and the same living environment as the matched patients were selected as the control group; and (3) all the subjects were not related and had not taken any drugs that could affect gastrointestinal function over the past 6 months. The exclusion criteria were as follows: (1) celiac disease; (2) hyperthyroidism, chronic pancreatitis or other diseases that could lead to changes in stool frequency and stool characteristics; (3) peptic ulcer or previous abdominal surgery; (4) history of hepatic or renal insufficiency; (5) serious diseases such as tumours; (6) mental illness, such as anxiety or depression; and (7) BMI (body mass index), routine blood tests, liver function, renal function, blood lipids and other indicators were not within the normal range. Select sample size calculation formula, n = $\frac{{\left( {t_{\alpha /2} + t_{\beta } } \right)^{2} \sigma^{2} }}{{\delta^{2} }}$ = 68 (α = 0.05, β = 0.10).

### Definition of risk factors for alcohol consumption

Women who drank more than one standard alcoholic unit per day and men who drank more than two standard alcoholic unit per day were considered to have a drinking risk factor. A standard alcoholic beverage was considered to contain 12–15 g of pure alcohol^22^.

### Laboratory analyses

Three millilitres of fasting venous blood was taken from all subjects in the morning and then anticoagulated with 600 μL ethylene diamine tetraacetic acid/tube. The collected samples were immediately stored in the refrigerator at – 80 ℃. A human genomic DNA kit was used to extract the DNA (Aidlab Biotechnologies Co., Ltd), and an ultramicro ultraviolet spectrophotometer nanodrop 2000 was used to detect the purity and concentration of DNA. The DNA sample concentration was between 12.5 and 200 g/μl, and the purity (A260/A280) was between 1.6 and 2.0; therefore, the samples could be used for polymerase chain reaction (PCR) detection (Tianjin Super Biotechnology Development Co., Ltd). SYBR was selected as the fluorescence channel for PCR amplification. Fluorescence quantitative PCR was used to collect fluorescence signals at wavelengths of 515–530 nm. The genotyping results were qualitatively interpreted according to the hole position shown in the result typing table. After PCR amplification, the Ct baseline threshold was set to 500 for result analysis. The inner reference hole was detected to form a good amplification curve CT value range (14 ≤ CT ≤ 25) and had a good characteristic melting curve. The peak value range of TM was between 81 and 86 ℃. A good amplification curve in the range of 14 ≤ CT ≤ 26 and a good fusion curve in the range of 80 ≤ TM ≤ 92 were considered positive pores. If the amplification curve or melting curve was not within the above range or there was no peak, it was considered a negative hole. The genotyping results were judged according to the result typing table in the specification of the specific positive hole query kit.

### Statistical analysis

SPSS 22.0 (SPSS, Inc., Chicago, Illinois, USA) was used for statistical analysis. Numerical data are expressed as the mean ± standard deviation (SD), while categorical variables are summarized as numbers (percentages). Cases with missing values were deleted. Paired logistic regression was used to analyse the risk factors for IBS. Potential risk factors for IBS were evaluated using univariate analyses, and the potential risk factors with P < 0.50 were included in the multivariate analysis. The relative risk was determined according to the odds ratio (OR) value and 95% confidence interval (95% CI). The interaction between genes and lifestyle habits in the context of the occurrence of IBS was analysed by logistic interaction analysis combined with an Excel table made by Andersson^23^. P < 0.05 was considered statistically significant.

## Results

### Subject characteristics

In this paired analysis, a total of 272 subjects were enrolled, including 136 IBS patients and 136 healthy controls. Among patients, the mean age was 20 ± 0.9 years, and 27.94% were men; among healthy controls, the mean age was 20 ± 1.4 years, and 27.94% were men. All of the subjects had completed the self-rated depression scale (SDS), and none of the subjects had anxiety or depression.

### Univariate and multivariate analyses of the risk factors for IBS

As shown in Table 1, HLA-DQ2, DQ4, DQ5, DQ6, DQ7, DQ8 and DQ9 were detected in both groups. Univariate analyses showed that the expression frequencies of HLA-DQ2 (14.7% vs. 29.4%, P = 0.039) and DQ8 (4.4% vs. 14.7%, P = 0.041) in the IBS group were significantly lower than those in the control group. In addition, univariate analyses showed that alcohol consumption might be a potential risk factor for IBS (27.9% vs. 10.3%, P = 0.009). Smoking; the consumption of tea, pickled products, fresh seafood, sour food, and spicy food; and other living habits were not significantly different between the IBS group and the control group. The risk factors with P < 0.5 in univariate analysis were included in paired logistic regression analysis. The results showed that the independent protective factors for IBS included HLA-DQ2 (OR: 0.262, 95% CI 0.087–0.787, P = 0.017) and HLA-DQ8 (OR: 0.189, 95% CI 0.045–0.800, P = 0.024), while alcohol consumption was an independent risk factor for IBS (OR: 7.000, 95% CI 1.591–30.800, P = 0.010), as shown in Table 2.

**Table1:** Univariate analysis of the risk factors for IBS.

Variables	IBS group (*n* = 136)	control group (*n* = 136)	χ^2^	P-value
DQ2, *n (%)*	20(14.7%)	40 (29.4%)	4.277	**0.039**
DQ4, *n (%)*	14 (10.3%)	10 (7.4%)	0.366	0.545
DQ5, *n (%)*	60 (44.1%)	60 (44.1%)	0.000	1.000
DQ6, *n (%)*	54 (39.7%)	40 (29.4%)	1.593	0.207
DQ7, *n (%)*	44 (32.4%)	34(25.0%)	0.899	0.343
DQ8, *n (%)*	6 (4.4%)	20 (14.7%)	4.168	**0.041**
DQ9, *n (%)*	24 (17.6%)	36(26.5%)	1.540	0.215
Smoking, *n (%)*	12 (8.8%)	6 (4.4%)	0.476	0.490
Consumption of alcohol, *n (%)*	38(27.9%)	14 (10.3%)	6.848	**0.009**
Consumption of tea, *n (%)*	74 (54.4%)	62 (45.6%)	1.059	0.303
Pickled products, *n (%)*	116 (85.3%)	106 (77.9%)	1.225	0.268
Fresh seafood, *n (%)*	106 (77.9%)	92 (67.6%)	1.819	0.177
Sour food, *n (%)*	118 (86.8%)	122 (89.7%)	0.283	0.595
Spicy food, *n (%)*	108 (79.4%)	112 (82.4%)	0.190	0.663

Values are presented as *n (%).*

Significant values are in bold.

**Table 2:** Multivariate analyses of the risk factors for IBS.

Variables	OR	95%CI	P-value
DQ2	0.262	0.087–0.787	**0.017**
DQ6	0.781	0.235–2.595	0.687
DQ7	0.570	0.160–2.030	0.385
DQ8	0.189	0.045–0.800	**0.024**
DQ9	0.134	0.015–1.196	0.072
Consumption of alcohol	7.000	1.591–30.800	**0.010**
Smoking	0.344	0.052–2.271	0.268
Consumption of tea	0.768	0.351–1.681	0.509
Pickled products	0.314	0.089–1.107	0.072
Fresh seafood	0.593	0.213–1.653	0.318

Significant values are in bold.

### Interaction between HLA-DQ2/DQ8 and alcohol consumption habits in the context of the occurrence of IBS

**Figure 1:**
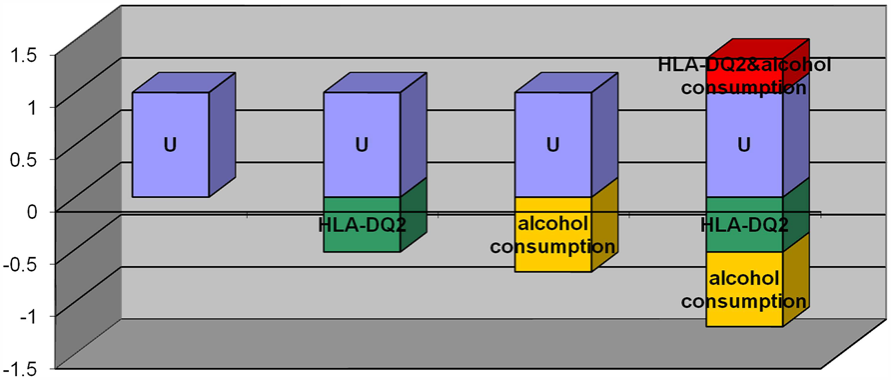
Interaction between HLA-DQ2 and alcohol consumption habits. Both HLA-DQ2 negative and alcohol consumption were assigned a value of 1; HLA-DQ2 positive, no alcohol consumption were assigned a value of 0; U was the control group, which represented the category carrying HLA-DQ2 and no alcohol consumption. Y-axis showed OR value. X-axis showed effects under different assignments.

**Figure 2:**
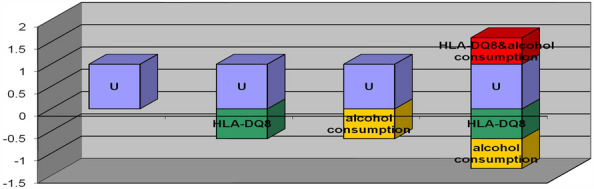
Interaction between HLA-DQ8 and alcohol consumption habits. Both HLA-DQ8 negative and alcohol consumption were assigned a value of 1; HLA-DQ8 positive, no alcohol consumption were assigned a value of 0; U was the control group, which represented the category carrying HLA-DQ8 and no alcohol consumption. Y-axis showed OR value. X-axis showed effects under different assignments.

Logistic interaction analysis showed that neither HLA-DQ2 nor HLA-DQ8 had a multiplicative interaction with alcohol consumption habits on the pathogenesis of IBS (P > 0.05), as shown in Table 3. After inputting the parameters into the Excel table made by Andersson, the CI of relative excess risk due to interaction (RERI) and attributable proportion (AP) for HLA-DQ2 and alcohol consumption habit contained 0 (Table 4 and Fig. 1). In contrast, the CI of RERI and AP for HLA-DQ8 and alcohol consumption habits was greater than 0, and the CI of the synergy index (S) was less than 1 (Table 5 and Fig. 2). The results showed that there was no additive interaction between HLA-DQ2 and alcohol consumption habits on the pathogenesis of IBS, while HLA-DQ8 and alcohol consumption habits had additive interactions in the context of the pathogenesis of IBS.

**Table 3:** Logistic interaction analysis for HLA-DQ2/DQ8 and alcohol consumption habits in the context of the occurrence of IBS.

Variables	β	P-value	OR (95%CI)
HLA-DQ2	− 0.742	0.422	0.476 (0.0782–2.916)
HLA-DQ8	− 1.101	0.110	0.333 (0.086–1.283)
Consumption of alcohol	− 1.253	0.039	0.286 (0.087–0.937)
HLA-DQ2 × consumption of alcohol	− 0.482	0.654	0.618 (0.075–5.095)
HLA-DQ8 × consumption of alcohol	− 1.351	0.055	0.259 (0.065–1.031)

**Table 4:** Additive interaction between HLA-DQ2 and alcohol consumption habits in the context of the occurrence of IBS.

	HLA-DQ2	alcohol consumption	HLA-DQ2& alcohol consumption
Regr. coefficients	− 0.74200	− 1.25300	− 2.47700
Cov HLA-DQ2	0.06848	0.04849	0.04865
Cov alcohol consumption	0.04849	0.11323	0.04925
Cov HLA-DQ2& alcohol consumption	0.04865	0.04925	0.06136

Exposure	RR	Lower	Upper
HLA-DQ2	0.476	0.285	0.795
alcohol consumption	0.286	0.148	0.552
HLA-DQ2& alcohol consumption	0.084	0.052	0.136

Measure	Estimate	Lower	Upper
RERI	0.322	− 0.029	0.674
AP	3.836	− 1.841	9.512
S	0.740	0.562	0.974

RERI: relative excess risk due to interaction; AP: attributable proportion; S: the synergy index.

**Table 5:** Additive interaction between HLA-DQ8 and alcohol consumption habits in the context of the occurrence of IBS.

	HLA-DQ8	alcohol consumption	HLA-DQ8& alcohol consumption
Regr. coefficients	− 1.10100	− 1.10200	− 1.35100
Cov HLA-DQ8	0.06848	0.04849	0.04865
Cov alcohol consumption	0.04849	0.11323	0.04925
Cov HLA-DQ8& alcohol consumption	0.04865	0.04925	0.06136

Exposure	RR	Lower	Upper
HLA-DQ8	0.333	0.199	0.555
alcohol consumption	0.332	0.172	0.642
HLA-DQ8& alcohol consumption	0.259	0.159	0.421

Measure	Estimate	Lower	Upper
RERI	0.594	0.331	0.858
AP	2.295	0.449	4.141
S	0.555	0.467	0.659

RERI: relative excess risk due to interaction; AP: attributable proportion; S: the synergy index.

## Discussion

This study revealed the risk factors for IBS in college students of Guangxi Han nationality. Multiple regression analysis showed that low expression of HLA-DQ2 and DQ8 was associated with the pathogenesis of IBS, while mild to moderate alcohol consumption was associated with the occurrence of IBS symptoms. Interaction analysis showed that in the context of the pathogenesis of IBS, HLA-DQ2 and alcohol consumption had neither multiplication nor additive effects, while HLA-DQ8 and alcohol consumption did not have multiplication effects but had additive effects. HLA-DQ8 and alcohol consumption have synergistic effects on the pathogenesis of IBS in Guangxi college students of Han nationality.

HLA is the first human genetic system that was found to have a clear relationship with many diseases. At present, it is the most complex and polymorphic genetic system in the human genome^24^. In recent years, some studies have supported that the IBS aetiology has a familial heredity component^14^. These studies mostly focused on the detection of the HLA-DQ2 and DQ8 genes, and the patients included mainly those with diarrhoea irritable bowel syndrome (IBS-D)^16,20^. To explore the relationship between the prevalence of IBS and the HLA-DQ gene in college students of Guangxi Han nationality, 136 IBS patients and 136 healthy controls were selected as the research subjects according to the principle of paired analysis. The results showed that the expression rates of the HLA-DQ2 gene (14.7% vs. 29.4%, P < 0.05) and HLA-DQ8 gene (4.4% vs. 14.7%, P < 0.05) in the IBS group were all lower than those in the control group. Multivariate analysis showed that the HLA-DQ2 gene (OR: 0.262, 95% CI 0.087–0.787, P = 0.017) and HLA-DQ8 gene (OR: 0.189, 95% CI 0.045–0.800, P = 0.024) were independent protective factors against IBS. Therefore, this study indicated that the HLA-DQ2 and DQ8 genes might be protective against IBS in college students of Guangxi Han nationality. That is, people with HLA-DQ2 or DQ8 had a low probability of IBS. This result is consistent with some previous studies. For example, Digiacomo et al. evaluated 443 patients in the gastroenterology clinic from southern Italy and found that 36.2% of IBS patients carried HLA-DQ2 or DQ8, which was significantly lower than the prevalence in the healthy control group^17^. Kårhus et al. screened 2293 subjects for HLA-DQ2 and DQ8 and found that the prevalence of IBS in patients without HLA-DQ2 or DQ8 genes was 4.6% (55/1197), while the prevalence of IBS in patients with HLA-DQ2 or DQ8 was only 2.6% (28/1086). They posited that carrying HLA-DQ2 or DQ8 genes seemed to reduce the prevalence of IBS^18^. However, there are also some disputes about the relationship between HLA-DQ2 or DQ8 and IBS. Domż ał–Magrowska et al. tested HLA-DQ2 and DQ8 in 48 IBS patients and 20 healthy controls in Poland. They found that the expression rate of HLA-DQ2 or DQ8 in IBS patients was 50%, while the expression rate of HLA-DQ2 was 20%, and no HLA-DQ8 gene was found in the healthy control group. They posited that HLA-DQ2 or DQ8 was the IBS susceptibility gene. A study from China also found that the HLA-DQB1 * 02 gene was a susceptibility gene in IBS-D patients of Han nationality in Guangdong, while the HLA-DQB1 * 0603 gene was a resistance gene^19^. These conclusions are contrary to those of previous studies in Italy and Denmark and our study. We speculate that the following reasons may explain the difference in conclusions. First, since HLA alleles are expressed differently in different geographical regions, there are some differences between susceptible genotypes and antagonistic genotypes of the same disease in different regions^25,26^. Second, the different research methods and typing methods used by researchers may also lead to some differences in the research results. In addition, the pathogenesis of IBS may be influenced by a combination of genetic and lifestyle factors. In fact, this conjecture was verified in a study by Aziz et al. They found that after six weeks of a gluten-free diet (GFD), 71% of IBS patients' symptom severity scores decreased by more than 50 points. Among them, subjects who were HLA-DQ2/8 negative showed a more significant reduction in abdominal distension symptoms^16^. A gluten-containing diet is common in European and American countries, and people with the HLA-DQ2 or DQ8 gene are likely to suffer from IBS if they consume a gluten-containing diet. However, most Chinese people, especially residents in Guangxi, consume rice as a staple food, which is different from a gluten-rich diet. Hence, research conclusions from European and American countries may not be applicable to individuals in Guangxi.

In the present study, univariate analyses and multivariate analysis results also showed that alcohol consumption was an independent risk factor for IBS in college students of Guangxi Han nationality (OR: 7.000, 95% CI 1.591–30.800, P = 0.010). This result was consistent with the results of many previous studies. For example, a study about the risk factors for IBS in Lebanese adults aged 18 to 65 years conducted by Chatila et al. showed that female sex, an age younger than 30 years old, regular hookah smoking and regular alcohol consumption were significantly associated with the high prevalence of IBS, and those who had ever consumed alcohol were twice as likely to suffer from IBS as those who had never consumed alcohol (P ˂ 0.01)^27^. A retrospective analysis conducted in Taiwan that included 56,355 patients with alcohol use disorder (AUD) and 225,420 randomly selected controls showed that the IBS risk of the AUD group was significantly higher than that of the control group. During the follow-up period, the incidence rate of IBS in AUD patients was 12.3 times that in non-AUD patients^28^. The reason may be that long-term alcohol intake affects the movement of the gastrointestinal tract, reduces the level of intestinal neuronal nitric oxide synthase (nNOS), reduces the nutrient absorption capacity of the intestines and changes the permeability of the intestinal mucosa. In addition, a large amount of alcohol will destroy the protective barrier of the intestinal mucosa, cause damage to the intestinal mucosa, reduce immunity, aggravate intestinal inflammation and stimulate the gastrointestinal tract. All of these effects can increase the burden on the gastrointestinal tract, causing symptoms such as dyspepsia, abdominal distension, abdominal pain and even diarrhoea^29,30^. Therefore, persuading IBS patients with alcoholism to greatly reduce their alcohol intake or even abstain from consuming alcohol according to their own circumstances and paying close attention to the improvement of symptoms after limiting alcohol intake is of great significance for the treatment and prevention of the recurrence of IBS.

As previously reported by Aziz et al., after a GFD, subjects who were HLA-DQ2/8 negative showed a more significant reduction in abdominal distension symptoms 16. Hence, we speculated that there was a certain correlation between HLA-DQ and dietary factors in the occurrence of IBS symptoms. To explore whether the pathogenesis of IBS could be influenced by a combination of genetics and diet, this study analysed the interaction between HLA-DQ2/DQ8 and alcohol consumption habits on the occurrence of IBS. In the analysis, the absence of HLA-DQ2 or DQ8 and alcohol consumption habits were assigned 1; otherwise, they were assigned 0. The results showed that the HLA-DQ2 gene and alcohol consumption habits had neither multiplicative interactions nor additive interactions in the context of the occurrence of IBS; that is, there was no interaction between them in the pathogenesis of IBS. Although the HLA-DQ8 gene and alcohol consumption habits had no multiplicative interaction on the occurrence of IBS (P > 0.05), after filling in the Excel table made by Andersson with parameters β 1, β 2, and β 3, the CIs of RERI and AP were all greater than 0, and the CI of S was less than 1, indicating that the HLA-DQ8 gene and alcohol consumption habit had additive interactions on the occurrence of IBS. The HLA-DQ8 gene and alcohol consumption habits had a synergistic effect on the occurrence of IBS. According to the results, this study indicated that if HLA-DQ8 gene-negative people had an alcohol consumption habit or even alcoholism, their risk of IBS would be higher. In addition, for IBS patients who do not carry the HLA-DQ8 gene but have a drinking habit, alcohol intake should be limited. These individuals could benefit from reducing their consumption of alcohol or abstinence.

Overall, this study suggested that the prevalence of IBS in college students of Guangxi Han nationality might be related to HLA-DQ gene polymorphisms. The HLA-DQ2 and HLA-DQ8 genes might be independent protective factors for IBS, and alcohol consumption might be an independent risk factor. The HLA-DQ2 gene and alcohol consumption habits had neither multiplicative nor additive interactions in the context of the occurrence of IBS, but there was a synergistic effect between HLA-DQ8 gene deletion and alcohol consumption habits in the context of the occurrence of IBS.

Nonetheless, this preliminary case‒control study also has some limitations. First, since we only enrolled college students of Guangxi Han nationality and the inclusion criteria included not taking any drugs over the past 6 months, our study included a relatively small cohort of patients. Second, our study did not cover other age groups, and the research conclusion was only applicable to college students of Guangxi Han nationality. Third, although we followed the case‒control principle in selecting research subjects, diet and genetics were not the only risk factors of IBS, and there might be other risk factors that we did not pay attention to involved in the occurrence of symptoms. Fourth, we confirmed through clinical diagnosis that the patients had IBS, but we did not continue to follow up to observe whether these patients would still have IBS if they did not drink alcohol. In the future, we will expand the sample size and the age group and expand the coverage of questionnaire surveys of the research subjects to further verify the research results. We will also continue follow-up to observe whether IBS persists after abstinence from alcohol in the patients.

## Data Availability

The datasets generated during and/or analyzed during the current study are available from the first author or corresponding author on reasonable request. All methods were carried out in accordance with relevant guidelines and regulations in methods section.
